# TTF-1- and/or CD56-positive Circulating Tumor Cells in patients with small cell lung cancer (SCLC)

**DOI:** 10.1038/srep45351

**Published:** 2017-03-28

**Authors:** Ippokratis Messaritakis, Dimitris Stoltidis, Athanasios Kotsakis, Eleftheria-Kleio Dermitzaki, Fillipos Koinis, Eleni Lagoudaki, Anastasios Koutsopoulos, Eleni Politaki, Stella Apostolaki, John Souglakos, Vassilis Georgoulias

**Affiliations:** 1 Laboratory of Tumor Cell Biology, School of Medicine, University of Crete, Crete, Greece; 2 Department of Medical Oncology, University General Hospital of Heraklion, Crete, Greece; 3 Pathology, University General Hospital of Heraklion, Crete, Greece

## Abstract

The aim of the study was to evaluate the phenotypic CTCs heterogeneity (TTF-1^+^ and/or CD56^+^) in SCLC patients and correlate it with the CellSearch. Peripheral blood was obtained from 108 consecutive patients. CTCs were detected by CellSearch and double-immunofluorescence using anti-CD45, anti-TTF-1 and anti-CD56 antibodies. Before chemotherapy TTF-1^+^/CD45^−^, CD56^+^/CD45^−^ and TTF-1^+^/CD56^+^ CTCs were detected in 66(61.1%), 55(50.9%) and 46(42.6%) patients, respectively; 60.2% of patients were CellSearch^+^. Among the 22 patients with 0 CTCs/7.5 ml on CellSearch, TTF-1^+^/CD45^−^, CD56^+^/CD45^−^ and TTF-1^+^/CD56^+^ CTCs were detected in 8(36.4%), 6(27.3) and 6(27.3%) patients, respectively; no CK^+^/EpCAM^+^ or TTF1^+^/EpCAM^+^ CTCs were detected in these patients. One-chemotherapy cycle decreased both the number of positive patients (*p* < 0.001) and their CTC number (*p* < 0.001), irrespectively of their phenotype and the detection method. The incidence and number of the different CTC subpopulations on PD, was significantly increased at their baseline levels. Multivariate analysis revealed that the increased number of CTCs at baseline and on PD were significantly associated with decreased PFS (*p* = 0.048) and OS (*p* = 0.041), respectively. There is an important CTC heterogeneity in such patients according to the expression of TTF-1 and CD56 which could detect EpCAM^−^ CTC subpopulations and, thus, undetectable by CellSearch. These CTC subpopulations are dynamically correlated with treatment efficacy and disease-progression.

Small Cell Lung Cancer (SCLC) is an aggressive disease that accounts for about 13% of lung cancer cases and almost two thirds of the patients present extensive-stage disease (ED-SCLC) at the time of presentation^1^,^2^. Front-line chemotherapy for ED-SCLC and chemo-radiotherapy for limited disease (LD-SCLC) are the standard recommended treatment which is associated with a high response rate; however, only 20–30% and 1–3% of patients with LD- and ED-SCLC, respectively, survive 5 years^3^,^4^.

The high metastatic potential of the disease is due to the dissemination of tumor cells through the hematogenous and/or the lymphatic vasculature. The detection of tumor cells in the peripheral blood (circulating tumor cells; CTCs) and bone marrow aspirates (disseminated tumor cells; DTCs) has been described in cancer patients^5^,^6^,^7^,^8^,^9^,^10^,^11^ and has been shown to be associated with shorter progression free (PFS) and overall (OS) survival in various tumor types, including SCLC^12^,^13^,^14^,^15^,^16^,^17^,^18^,^19^,^20^. In SCLC patients, the detection of CTCs before the initiation of systemic treatment as well as post-treatment and at the time of clinical relapse has been shown to be associated with a worse overall survival^15^,^17^,^18^,^19^,^20^. Moreover, Hou *et al*.^15^, using the cell size and filtration (ISET) platform, described the presence of circulating tumor microemboli (CTM) which were also associated with unfavorable clinical outcome. More importantly, they reported that a substantial proportion of the solitary CTCs but none of the CTCs comprising to CTMs were apoptotic^15^.

SCLC cells are characterized by the expression of various neuroendocrine peptides, such as CD56 (NCAM), chromogranin A (CgA) and synaptophysin^21^,^22^,^23^. In addition, TTF-1, which can be detected in different histologic subtypes of lung carcinomas^24^,^25^,^26^,^27^, is also expressed in almost 85–90% of SCLC tumor cells^22^; moreover, CD56 (NCAM), a cell surface sialoglycoprotein, is specifically expressed in neuroendocrine tissues and tumors, including SCLC^28^,^29^. TTF-1 is a transcription protein of the Nkx-2 gene family and regulates the early human development, cell growth and morphogenesis of thyroid, lung and restricted areas of diencephalon^30^.

The investigation of the phenotype of CTCs is an interesting topic since accumulating evidence points to functional heterogeneity within the CTC compartment with possible clinical implications. Indeed, it has been previously reported that CTCs in SCLC patients are heterogeneous based on their apoptotic and proliferative phenotype^15^. An important problem concerning the investigation of CTCs is related to the used detection methods. Indeed, the clinically validated CellSearch (CS) platform is based on the immunomagnetic capture of EpCAM-positive CTCs which, subsequently, are stained with an anti-pancytokeratin antibody and DAPI; however, since CTCs undergoing Epithelial-Mesenchymal-Transition (EMT) down-modulate the epithelial markers, including EpCAM, it is obvious that CS fails to capture EpCAM-negative CTCs. Therefore, it is interesting to further evaluate the heterogeneity of CTCs in patients with SCLC using known and well established markers, such as TTF-1 and CD56 which are expressed on neuroendoctrine tumor cells. In addition, the presence of CD56- and/or TTF-1-positive CTCs could offer the opportunity to detect CTCs which are undetectable by CS. The aim of the present study was to investigate the phenotypic heterogeneity of CTCs in SCLC patients using the TTF-1 and CD56 neuroendocrine markers.

## Patients and Methods

### Patient samples and cytospin preparations

Peripheral blood (20 ml in EDTA and 7.5 ml in CellSearch Save preservative tubes; Raritan, NJ, USA) was obtained from newly-diagnosed patients with SCLC before the initiation of systemic treatment (n = 108), after the administration of one chemotherapy cycle (n = 76) and at the time of disease progression (PD; n = 48). All blood samples were obtained at the middle-of-vein puncture after the first 5 ml were discarded to avoid contamination with epithelial cells from the skin. All patients had histologically confirmed SCLC. Patients with ED-SCLC received up to 6-cycles of etoposide/cisplatin while patients with LD-SCLC were treated with concurrent etoposide/cisplatin and radiotherapy. The study has been approved by the Ethics and Scientific Committees of the University General Hospital of Heraklion and all patients signed a written informed consent. All methods were performed in accordance with the relevant guidelines and regulations.

Peripheral blood mononuclear cells (PBMCs) were isolated by Ficoll–Hypaque density gradient (*d* = 1,077 g/ml; Sigma-Aldrich, GmbH, Germany) centrifugation at 1,800 rpm for 30 min. Centrifugation and cytospins were prepared as previously described^31^,^32^. In brief, aliquots of 5 × 10^5^ PBMCs were cyto-centrifuged at 2,000 rpm for 2 min on glass microscope slides. Cytospins were air dried and stored at −80 °C until use. Two slides (10 × 10^5^ PBMCs) from each patient were analyzed at each time point.

### Detection of CTCs using the CS platform

For the enumeration of CTCs, peripheral blood samples maintained at ambient temperature and processed within 72 h using the CS platform (Veridex LCC, Raritan, NJ) according to the manufacturer’s instructions. CTC morphology was confirmed in all cases and analysis was performed with the CellTracks Analyser II by experienced biologists (E.P and S.A.). Results are expressed as number of CTCs/7.5 ml blood.

### Double Immunofluorescence Assay (IF)

TTF-1 and/or CD56 were detected by double IF staining using monoclonal antibodies either against TTF-1 (DAKO, Agilent Technologies, Denmark) or CD56 (NCAM) (Thermo Fisher Scientific, Fremont, CA, USA) and anti-CD45 (Common Leukocyte Antigen; Santa Cruz, CA, USA) as previously described^33^. Moreover, cytospins were double stained with the mouse anti-TTF-1 or anti-A45-B/B3 (anti-cytokeratins 8, 18, and 19; Micromet, Munich, Germany) and the mouse anti-EpCAM (Acris Antibodies GmbH, Germany) antibodies. In brief, the incubation period for all primary and secondary antibodies was 1 h. Zenon technology (FITC-conjugated IGg1 antibody; Molecular Probes, Invitrogen, CA, USA) was used for the TTF-1 and A45-B/B5 detection. CD56 was labelled with Alexa 555 or Alexa 488 (Molecular Probes); EpCAM and CD45 were labelled with Alexa 555. The omission of the first antibodies has been used as negative controls. DAPI-antifade (Molecular Probes) was added to each sample for nuclear staining. Slides were analyzed using a fluorescence microscope (Leica DM 2500, Heidelberg, Germany). Results are expressed as number of CTCs/10^6^ PBMCs.

### Cell Line

The human SCLC cell line, H209 (ATCC^®^ HTB-172) was obtained from the American Type Culture Collection (Manassas, VA, USA) and used as positive control in IF experiments. H209 cells were cultured in RPMI 1640 (Gibco-BRL Life Technologies, Grand Island, NY), supplemented with 10% fetal bovine serum (Gibco-BRL), 2 mM L-glutamine (Gibco-BRL), 10 mMHepes (Gibco-BRL), 1 mM sodium pyruvate (Gibco-BRL), 1.5 g/L NaHCO_3_ (Sigma-Aldrich), 4.5 g/L glucose (Sigma-Aldrich) and 50 mg/ml penicillin/streptomycin (Gibco-BRL). Cells were maintained in a humidified atmosphere of 5% CO_2_, at 37 °C. To determine the sensitivity of the method, H209 cells were spiked in peripheral blood obtained from healthy individuals, and the PBMCs and the corresponding, obtained after Ficoll-Hypaque density centrifugation, and cytospins were prepared as above. All experiments were performed during the logarithmic growth phase of the cell line.

### Study design and statistics

This is a prospective, single institution study, investigating the expression of CD56 and TTF-1 in CTCs from consecutive patients with SCLC. There was no specific statistical design since the aims of the study were to characterize the heterogeneity of CTCs and to define the presence of phenotypically different subpopulations of CTCs during front-line treatment. The evaluation of CTCs was done blindly to clinical data. PFS was calculated for all patients from the date of the first treatment administration until treatment discontinuation due to disease progression or death. OS was calculated for all patients from enrolment in the study to the date of death due to any cause. The association of risk factors with PFS and OS was analyzed using the log rank test and the Kaplan–Meier method. Univariate and multivariate Cox proportional hazards regression models with hazard ratios (HR) and 95% CIs were used to evaluate the predictive and prognostic relevance of the CTCs. Statistical significance was set at *p* = 0.05. Statistical analysis was performed using the SPSS v. 20 software (IBM Corp. Armonk, NY, USA).

## Results

### CD56 and TTF-1 expression in H209 cells and PBMCs of healthy individuals

Immunofluorescent staining (IF) revealed that 85% ± 5%, 85% ± 15% and 68% ± 5% /10^6^ H209 cells were TTF-1^+^, CD56^+^ and TTF-1^+^/CD56^+^, respectively (mean +/−SD values from 5 experiments) (Supplementary Table S1). Spiking experiments with 1–1000 H209 cells/10^6^ PBMCs from blood donors demonstrated that IF could detect up to 1 H209 cell/10^6^ PBMCs. Control experiments performed in 26 healthy blood donors revealed that 15 ± 4%/10^6^ PBMCs were CD56^+^ but all of them were also CD45^+^. Conversely, no TTF-1^+^/CD45^−^, TTF-1^+^/CD45^+^ or TTF-1^+^/CD56^+^ cells could be detected in normal PBMCs (data not shown).

### Patients’ characteristics

From 11/2010 to 05/2015, 108 consecutive patients with SCLC were enrolled in the study. The patients’ characteristics are listed in Table 1. The median age was 66 years, 91 (84.3%) patients were males, 63 (58.3%) had PS (ECOG) 0–1, and 71 (65.7%) had ED-SCLC. Moreover, 79 (73.1%) patients had lactate dehydrogenase (LDH) serum levels above the upper normal limit, 12 (11.1%) had brain, 40 (37.0%) liver and 32 (29.6%) bone metastases at the time of diagnosis. Thirty eight (35.2%) patients received concurrent chemo-radiotherapy for LD-SCLC and 77 (71.3%) achieved an objective response (CR/PR) (Table 1). Eighty-three (76.9%) patients were evaluable for CTC enumeration using the CS before treatment initiation whereas in 25 patients, the failure to enumerate CTCs at baseline was due to various reasons (Supplementary Figure S1). CTCs (≥5 CTCs/7.5 ml) could be detected in 50 (60.2%) patients with a median number of 14 CTCs/7.5 ml of blood (range, 0–10.000) (Table 2). The detection of CTCs was correlated with patients’ PS (*p* = 0.001), disease stage (*p* < 0.001), concurrent chemo-radiotherapy in LD-SCLC (*p* < 0.001), LDH levels (*p* = 0.001), liver or bone metastases (*p* < 0.001) and response to treatment (*p* = 0.034) (Table 1).

### Detection of TTF-1^+^/CD45^−^, CD56^+^/CD45^−^ and TTF-1^+^/CD56^+^ CTCs before treatment initiation

**Figure 1:**
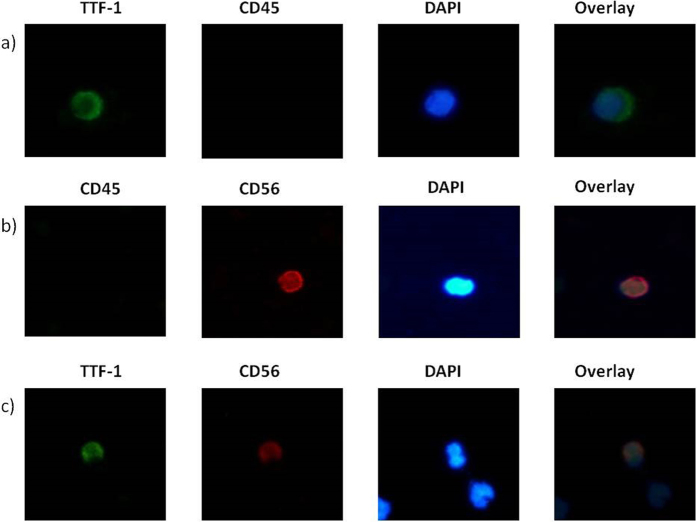
TTF1(+)/CD45(−), CD56(+)/CD45(−) and TTF1(+)/CD56(+) CTCs by double immunofluorescent staining.

**Figure 2:**
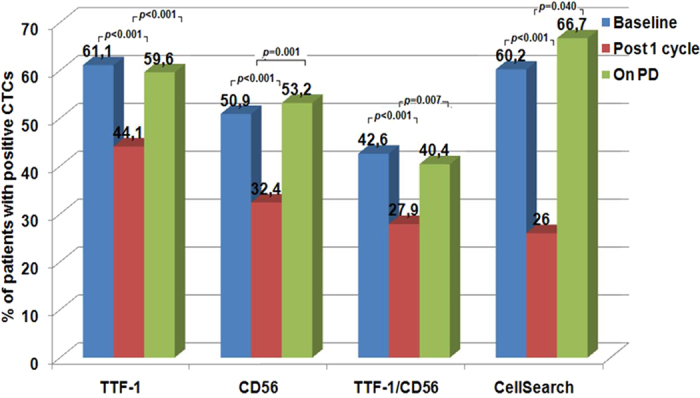
Detection of CTCs in patients with SCLC patients during front line treatment.

At baseline, IF demonstrated the presence of TTF-1^+^/CD45^−^, CD56^+^/CD45^−^ and TTF-1^+^/CD56^+^ (Fig. 1). Sixty-six (61.1%) patients had TTF-1^+^/CD45^−^ CTCs, whilst 55 (50.9%) and 46 (42.6%) had CD56^+^/CD45^−^ and TTF-1^+^/CD56^+^ CTCs; 37 (34.3%) patients had undetectable CTCs by IF (Table 2 and Fig. 2). There was a significant association between the detection of the different subpopulations of CTCs and the presence of liver (TTF-1^+^/CD45^−^, *p* = 0.027; CD56^+^/CD45^−^, *p* = 0.036) or bone (TTF-1^+^/CD45^−^, *p* = 0.041; TTF-1^+^/CD56^+^, *p* = 0.006) metastases (Table 1). The median number of TTF-1^+^/CD45^−^, CD56^+^/CD45^−^ and TTF-1^+^/CD56^+^ CTCs was 3/10^6^ PBMCs (range, 0–169), 1/10^6^ PBMCs (range, 0–940) and, 0/10^6^ PBMCs (range, 0–75), respectively (Table 2).

Thirty-five (70.0%), 27 (54.0%) and 25 (50.0%) out of 50 patients with detectable (≥5 CTCs/7.5 ml) CTCs at baseline by CS, had also detectable TTF-1^+^/CD45^−^, CD56^+^/CD45^−^ and TTF-1^+^/CD56^+^ CTCs, respectively. There was a significant correlation between the detection of CTCs by CS at baseline and the TTF-1^+^/CD45^−^ (*p* = 0.03) and TTF-1^+^/CD56^+^ (*p* = 0.049) but not the CD56^+^/CD45^−^ subpopulations (Table 3). TTF-1^+^/CD45^−^, CD56^+^/CD45^−^ and TTF-1^+^CD56^+^ CTCs could also be detected in patients with <5 CTCs/7.5 ml of blood (Table 3).

### Detection of TTF-1^+^/CD45^−^, CD56^+^/CD45^−^ and TTF-1^+^/CD56^+^ CTCs after one chemotherapy cycle and at relapse

In 76 (70.4%) patients, a second blood sample was obtained after the first treatment cycle. As shown in Fig. 2, the number of patients with detectable CTCs was significantly decreased compared to the corresponding baseline values. TTF-1^+^/CD45^−^, CD56^+^/CD45^−^ and TTF-1^+^/CD56^+^ cells were detected in 30 (44.1%), 22 (32.4%) and 19 (27.9%) patients, respectively by IF whereas ≥5 CTCs/7.5 ml of blood was detected in 16 (29.1%) patients. Chemotherapy also resulted in a significant decrease of the number of TTF-1^+^/CD45^−^ (*p* < 0.001), CD56^+^/CD45^−^ (*p* < 0.001) and TTF-1^+^/CD56^+^ (*p* < 0.001) cells, respectively, as well as of CTCs detected by CS (*p* < 0.001), compared to baseline values (Table 2). TTF-1^+^/CD45^−^, CD56^+^/CD45^−^ or TTF-1^+^/CD56^+^ CTCs could be detected by IF both in patients with ≥5 CTCs/7.5 ml of blood and in patients with <5 CTCs/7.5 ml of blood (Table 3). Figure 2 also indicates that the number of patients with detectable CTCs by IF (TTF-1^+^/CD45^−^, CD56^+^/CD45^−^,TTF-1^+^/CD56^+^) or by CS, was significantly increased on PD compared to that after one treatment cycle. Moreover, the median number of TTF-1^+^/CD45^−^, CD56^+^/CD45^−^, and TTF-1^+^/CD56^+^ CTCs was significantly increased on PD (Table 2) both in the group of patients with ≥5 CTCs/7.5 ml of blood and <5 CTCs/7.5 ml of blood (Table 3).

### Detection of CTC subpopulations in patients without detectable CTCs by CS

In 22 patients no CTCs could be detected by CS (0 CTCs/7.5 ml of blood) (Table 3). However, IF revealed the presence of TTF-1^+^/CD45^−^, CD56^+^/CD45^−^ and TTF-1^+^/CD56^+^ CTCs in eight (36.4%), six (27.3%) and six (27.3%) patients, respectively (Table 3). Table 4 demonstrates that in 6 out of 8 patients with detectable CTCs by IF but not by CS, all the subpopulations of CTCs were present; in addition, IF revealed that these patients did not have detectable TTF-1^+^/EpCAM^+^ or CK^+^/EpCAM^+^ CTCs. Ιn addition, no CK^+^/EpCAM^+^ CTCs could be detected in the remaining 14 patients without detectable CTCs by CS (data not shown). Similarly, the phenotypically different CTC subpopulations could be detected in patients without detectable CTCs both after one treatment cycle and on PD (Table 3).

### Detection of CTC subpopulations and clinical outcome

**Figure 3:**
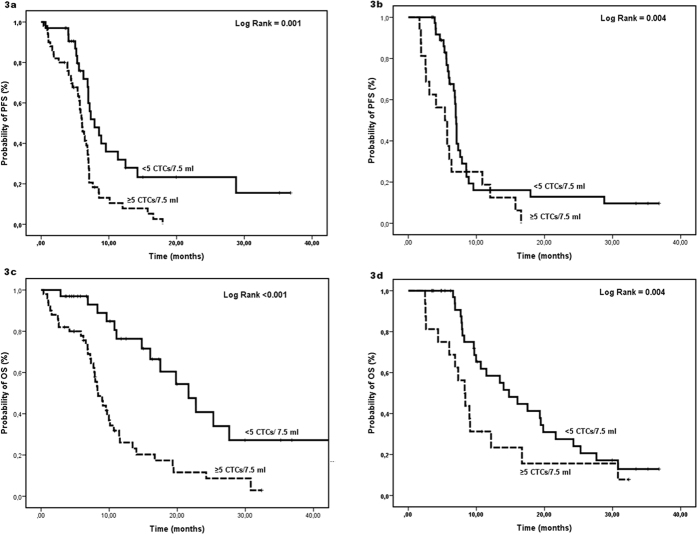
Kaplan Meier curves for PFS and OS according to the detection of CTCs by CS. PFS: (**a**) at baseline; (**b**) after one-cycle of treatment. OS (**c**) at baseline; (**d**) after one-cycle of treatment.

Clinical relapse was observed in 89 (82.4%) patients. The incidence of detection of a high number of CTCs by CS both at baseline and after one treatment cycle was higher in the group of patients who experienced a PD compared to patients with no PD (54.2% vs 6.0%; *p* = 0.004 and 29% vs 0.0%; *p* = 0.022); however, using IF this difference could not reach any statistical significance (Supplementary Table S2).The median PFS for the whole group of patients was 6.8 months (95% CI: 6.2–7.5). In patients with and without detectable CTCs by CS at baseline, the median PFS was 6.0 and 7.9 (95% CI: 5.4–6.7 and 5.7–10.1) months, respectively (*p* = 0.001; Fig. 3a); the median PFS was also significantly shorter in patients with detectable CTCs after one chemotherapy cycle (*p* = 0.004; Fig. 3b; Supplementary Table S3). PFS could not reach any statistical significance according to the different CTC subpopulations either at baseline or after one treatment cycle. The median OS for the whole group of patients was 10.8 months (95% CI: 8.8–12.8). In patients with and without detectable CTCs by CS at baseline, the median OS was 8.4 and 21.7 (95% CI: 7.0–9.8 and 15.6–27.7) months, respectively (*p* < 0.001; Fig. 3c). In addition, the median OS was significantly different in patients with and without detectable CTCs either after one chemotherapy cycle (*p* = 0.004; Fig. 3d) or on PD (*p* = 0.021; Supplementary Table S3).

### Univariate and Multivariate Analysis

Univariate analysis revealed that PS (ECOG), disease stage, LDH levels, organ metastases, response to treatment and increased number of CTCs at baseline and after one treatment cycle of were significantly associated with a shorter PFS (Supplementary Table S4). Multivariate analysis showed that increased number of CTCs at baseline could be emerged as independent factor associated with a reduced PFS (HR: 1.9, 95% CI: 0.9–3.9; *p* = 0.048) (Supplementary Table S4).

Similarly, PS (ECOG), disease stage, LDH levels, organ metastases, response to treatment and increased number of CTCs at baseline and at the time of PD were significantly associated with a shorter OS in univariate analysis (Supplementary Table S4). Again, in multivariate analysis only the increased number of CTCs at the time of PD could be emerged as independent prognostic factor associated with a decreased OS (HR: 2.1, 95% CI: 0.9–5.3; *p* = 0.041) (Supplementary Table S4).

## Discussion

The prognostic value of CTCs has been previously reported by several investigators^15^,^17^. Indeed, Hou *et al*^15^. reported that CTCs were present in 85% of the patients before chemotherapy and their number (≥50 CTCs/7.5 ml of blood) was associated with a decreased median PFS and OS^15^. Similarly, Shi *et al*., using a molecular assay for the detection of CTCs, also demonstrated that CTCs detection represent a high risk for both reduced PFS and OS in patients with SCLC^34^.

The current study, demonstrates for the first time that CTCs from patients with newly diagnosed SCLC could be detected using the TTF-1 and CD56 molecules which are expressed in tumor cells of neuroendocrine origin since both TTF-1 and CD56 are expressed in 89–100%^21^,^24^,^27^,^35^ and in (90–99%)^36^, of SCLC cases, respectively. In addition, the detection of TTF-1^+^ and/or CD56^+^ CTCs clearly revealed the phenotypic heterogeneity of CTCs in patients with SCLC as well as their changes during the different clinical phases of the disease. Indeed, TTF-1^+^/CD45^−^, CD56^+^/CD45^−^ and TTF-1^+^/CD56^+^ CTCs could be detected in patients before the initiation of any systemic treatment, after one-cycle and at the time of PD. However, in almost 10–15% of SCLC cases, no TTF-1 expression can be detected^37^,^38^,^39^, indicating the absence of these neuroendocrine markers in some CTC subpopulations. On the contrary, the detection of TTF-1^+^/CD56^+^ CTCs, which represent a minority among the whole population of CTCs since their number was less than that of TTF-1^+^ or CD56^+^, further supports the heterogeneity of CTCs in patients with SCLC. Indeed, previous studies have reported both inter- and intra-patient’s CTCs heterogeneity by using different methodologies and tumor types^40^,^41^,^42^,^43^. Whether this phenotype represents a specific step of the malignant cell’s differentiation or a particular genetic change is unknown.

The presented results also demonstrate that even one treatment cycle resulted to a significant decrease not only of the number of patients with ≥1 CTCs/10^6^ PBMCs but also of their absolute number; conversely, on disease progression both the number of patients with detectable CTCs as well as the number of CTCs were significantly increased reaching, practically, their baseline levels. These dynamic changes were observed irrespectively of the phenotype of CTC subpopulation. It is interesting to note that in the majority of patients, chemotherapy could not completely eliminate the different CTC subpopulations suggesting that some of them are chemo-sensitive whereas others seem to be chemo-resistant. Similar findings have been observed in other tumor types^44^,^45^,^46^,^47^,^48^. Moreover, Hou *et al*. (2012) observed an increase in CTCs expressing the anti-apoptotic protein Bcl-2 after chemotherapy, thus pointing the usefulness of CTCs stratification and pharmacodynamic monitoring in trials^15^. Additionally, they found that the presence of apoptotic CTCs at baseline is significantly correlated with disease stage and number of metastatic sites^15^, whilst intra- and inter-patient heterogeneity was observed for EMT markers in both CTCs and circulating tumor microemboli (CTMs)^49^. Furthermore, the genetic heterogeneity has been documented by single cell genomic hybridization, on DTCs^50^. These observations further support both the phenotypic and molecular heterogeneity of CTCs/DTCs in SCLC patients^51^,^52^,^53^.

The presented results also demonstrated for the first time that in a subgroup of patients, CS failed to detect CTCs despite their detection by IF. This observation should be attributed to the finding that, in these particular patients, IF staining could not reveal the presence of either TTF-1^+^/EpCAM^+^ or CK^+^/EpCAM^+^ CTCs since CS recognizes CK^+^ CTCs which are, previously, captured by an anti-EpCAM antibody. It is well known that epithelial tumor cells migrating through the bloodstream undergo EMT which is characterized, among others, by a loss of expression of epithelial markers^32^,^54^,^55^; therefore, it is reasonable to hypothesize that, in this small subgroup of patients without detectable CTCs by CS, the TTF-1^+^/EpCAM^−^ CTCs probably represent CTCs undergoing EMT. This is in accordance with studies reporting that the CTC detection rate was higher using EpCAM-independent enrichment methods^56^. Thus, a combination of both EpCAM- and non-EpCAM-based CTC technologies seems to be needed in order to better assess the presence of CTCs in patients with SCLC and to evaluate their biological and clinical relevance. It should be noted that new and promising methodologies have also been developed for the detection of CTCs based on nanoparticles^57^,^58^,^59^,^60^,^61^,^62^; however, these methods have serious limitations, such as the higher cost and the requirement of special equipment compared to the simple immunofluorescence staining.

Previous studies using the CS platform have shown that the detection of CTCs either before the initiation of systemic treatment or after one chemotherapy cycle is significantly associated with PFS and OS^15^,^17^. However, a recent study reported that although CTCs have a useful prognostic role at baseline, only the important reduction in the CTC number after one chemotherapy cycle significantly improved prognostic accuracy^63^. Our findings are in agreement with the above reports; however, there was no significant correlation between the different CTC subpopulation and the patients’ clinical outcome. There is no clear explanation for this observation; however, we cannot exclude that the expression of TTF-1 and CD56 molecules in CTCs are not related with the invasive potential or the aggressivity of tumor cells in contrast with the more epithelial EpCAM^+^/CK^+^ CTCs detected by CS. Alternatively, we cannot exclude that the used discrimination cut-off in order to characterize the positive samples could be related to this observation; it should be noted that Hou *et al*.^15^, used as cut-off the 50 CTC/7.5 ml of blood for their analysis.

In conclusion, the current study demonstrated an important phenotypic heterogeneity of CTCs in patients with SCLC and the different subpopulations of CTCs could be used as potential dynamic biomarkers during the different clinical phases of the disease. Subsequent studies should evaluate the biological relevance of these phenotypically different subpopulations of CTCs and, especially, of EpCAM-negative CTCs as well as whether their specific molecular and/or genetic profile could provide information towards a more individualized treatment.

## Additional Information

**How to cite this article:** Messaritakis, I. *et al*. TTF-1- and/or CD56-positive Circulating Tumor Cells in patients with small cell lung cancer (SCLC). *Sci. Rep.* **7**, 45351; doi: 10.1038/srep45351 (2017).

**Publisher's note:** Springer Nature remains neutral with regard to jurisdictional claims in published maps and institutional affiliations.

## Supplementary Material

Supplementary Information

## Figures and Tables

**Table 1:** Clinical characteristics of SCLC patients.

	N (%)	TTF-1 (%) (N = 108)		*p*	CD56 (%) (N = 108)		*p*	TTF-1/CD56 (%) (N = 108)		*p*	CellSearch (%) ≥5CTCs (N = 83)		*p*
		+ve	−ve		+ve	−ve		+ve	−ve		−ve	≥5 CTCs	
Age	Median 66 (range, 44–82)												
Gender													
Male	91 (84,3%)	58 (53,7%)	33 (30,6%)	0,153	46 (42,6%)	45 (41,7%)	0,53	39 (36,1%)	52 (48,1%)	0,56	42 (50,6%)	28 (33,7%)	0,586
Female	17 (15,7%)	8 (7,4%)	9 (8,3%)		9 (8,3%)	8 (7,4%)		7 (6,5%)	10 (9,3%)		8 (9,6%)	5 (6,0%)	
PS													
0–1	63 (58,3%)	38 (35,2%)	25 (23,1%)	0,501	31 (28,7%)	32 (29,6%)	0,41	28 (25,9%)	35 (32,4%)	0,4	23 (27,7%)	27 (32,5%)	0,001
≥2	45 (41,7%)	28 (25,9%)	17 (15,7%)		24 (22,2%)	21 (19,4%)		18 (16,7%)	27 (25,0%)		27 (32,5%)	6 (7,2%)	
Stage													
LD	37 (34,3%)	22 (20,4%)	15 (13,9%)	0,48	17 (15,7%)	20 (18,5%)	0,29	14 (13,0%)	23 (21,3%)	0,3	8 (9,6%)	21 (25,3%)	<0,001
ED	71 (65,7%)	44 (40,7%)	27 (25,0%)		38 (35,2%)	33 (30,6%)		32 (29,6%)	39 (36,1%)		42 (50,6%)	12 (14,5%)	
LD wih RT													
Yes	38 (35,2%)	22 (20,4%)	16 (14,8%)	0,381	17 (15,7%)	21 (19,4%)	0,23	14 (13,0%)	24 (22,2%)	0,25	8 (9,6%)	22 (26,5%)	<0,001
No	70 (64,8%)	44 (40,7%)	26 (24,1%)		38 (35,2%)	32 (29,6%)		32 (29,6%)	35 (35,2%)		(42 (50,6%)	11 (13,3%)	
LDH													
High	79 (73,1%)	51 (48,1%)	28 (26,4%)	0,242	41 (38,7%)	38 (35,8%)	0,46	33 (31,1%)	46 (43,4%)	0,36	43 (52,4%)	17 (20,7%)	0,001
Low	27 (25,0%)	14 (13,2%)	13 (12,3%)		13 (12,3%)	14 (13,2%)		13 (12,3%)	14 (13,2%)		7 (8,5%)	15 (18,3%)	
Unknown	2 (1,9%)	1 (0,9%)	1 (0,9%)		1 (0,9%)	1 (0,9%)		1 (0,9%)	1 (0,9%)		—	—	
Liver Metastases													
Yes	40 (37,0%)	29 (26,9%)	11 (10,2%)	0,027	25 (23,1%)	15 (13,9%)	0,036	21 (19,4%)	19 (17,6%)	0,07	28 (33,7%)	3 (3,6%)	<0,001
No	66 (61,1%)	37 (34,3%)	29 (26,9%)		30 (27,8%)	36 (33,3%)		25 (23,1%)	41 (38,0%)		21 (25,3%)	30 (36,1%)	
Unknown	2 (1,9%)	0 (0,0%)	2 (1,9%)		0 (0,0%)	2 (1,9%)		0 (0,0%)	2 (1,9%)		1 (1,2%)	0 (0,0%)	
CNS at diagnosis													
Yes	12 (11,1%)	7 (6,5%)	5 (4,6%)	0,563	6 (5,6%)	6 (5,6%)	0,59	5 (4,6%)	7 (6,5%)	0,41	6 (7,2%)	4 (4,8%)	0,787
No	92 (85,2%)	58 (53,7%)	34 (31,5%)		46 (42,6%)	46 (42,6%)		41 (38,0%)	51 (47,2%)		43 (51,8%)	29 (34,9%)	
Unknown	4 (3,7%)	1 (0,9%)	3 (2,8%)		3 (2,8%)	1 (0,9%)		0 (0,0%)	4 (3,7%)		1 (1,2%)	0 (0,0%)	
Bone Metastases													
Yes	32 (29,6%)	23 (21,3%)	9 (8,3%)	0,041	20 (18,5%)	12 (11,1%)	0,06	19 (17,6%)	13 (12,0%)	0,006	23 (27,7%)	1 (1,2%)	<0,001
No	71 (65,7%)	42 (38,9%)	29 (26,9%)		34 (31,5%)	37 (34,3%)		27 (25,0%)	44 (40,7%)		25 (30,1%)	30 (36,1%)	
Unknown	5 (4,6%)	1 (0,9%)	4 (3,7%)		1 (0,9%)	4 (3,7%)		0 (0,0%)	5 (4,6%)		2 (2,4%)	2 (2,4%)	
Response													
CR/PR	77 (71,3%)	46 (42,6%)	31 (28,7%)	0,79	38 (35,2%)	39 (36,1%)	0,86	33 (30,6%)	44 (40,7%)	0,48	32 (38,6%)	28 (33,7%)	0,034
SD	11 (10,2%)	7 (6,5%)	4 (3,7%)		6 (5,6%)	5 (4,6%)		6 (5,6%)	5 (4,6%)		6 (7,2%)	4 (4,8%)	
PD	14 (13,0%)	10 (9,3%)	4 (3,7%)		7 (6,5%)	7 (6,5%)		6 (5,6%)	8 (7,4%)		9 (10,8%)	1 (1,2%)	
Unknown	6 (5,6%)	3 (2,8%)	3 (2,8%)		4 (3,7%)	2 (1,9%)		1 (0,9%)	5 (4,6%)		3 (3,6%)	0 (0,0%)	
Relapse													
Yes	89 (82,4%)	54 (50,0%)	35 (32,4%)	0,84	46 (42,6%)	43 (39,8%)	0,73	37 (34,3%)	52 (48,1%)	0,64	45 (54,2%)	21 (25,3%)	0,004
No	19 (17,6%)	12 (11,1%)	7 (6,5%)		9 (8,3%)	10 (9,3%)		9 (8,3%)	10 (9,3%)		5 (6,0%)	12 (14,5%)	

**Table 2:** Detection of different subpopulations of CTCs during treatment.

	Baseline		After one treatment cycle		At disease progression	
	N (%)	Median (range)	N (%)	Median (range)	N (%)	Median (range)
TTF-1^+^/CD45^−^	66/108 (61,1)	3 (0–169)	30/76 (44,1)*	0 (0–56)	28/48 (59,6)^a^	2 (0–149)
CD56^+^/CD45^−^	55/108 (50,9)	1 (0–940)	22/76 (32,4)**	0 (0–67)	25/48 (53,2)^b^	2 (2 (0–246)
TTF-1^+^/CD56^−^	46/108 (42,6)	0 (0–75)	19/76 (27,9)***	0 (0–25)	19/48 (40,4)^c^	0 (0–70)
CellSearch	50/83 (60,2)	14 (0–10000)	13/50 (26,0)****	0 (0–1464)	24/36 (66,7)^d^	43 (0–11143)

*p* value: Baseline vs Post 1st: * < 0,001; ** < 0,001; *** < 0,001; **** < 0,001. *p* value: Post 1st vs Progression: ^a^ < 0,001; ^b^0,001; ^c^0,007; ^d^0,04

**Table 3:** Detection of different subpopulations of CTCs by IF in patients without detectable CTCs by CS.

CTCs /7,5 ml of blood	Baseline			After one treatment cycle			At disease progression		
	TTF-1^+^/ CD45^−^	CD56^+^/ CD45^−^	TTF-1^+^/ CD56^+^	TTF-1^+^/ CD45^−^	CD56^+^/ CD45^−^	TTF-1^+^/ CD56^+^	TTF-1^+^/CD45^−^	CD56^+^/CD45^−^	TTF-1^+^/CD56^+^
**≥5 CTCs**	35/50 (70,0%)	27/50 (54,0%)	25/50 (50,0%)	7/13 (53,8%)*	4/13 (30,8%)	4/13 (30,8%)**	14/24 (58,3%)	12/24 (50,0%)	10/24 (41,7%)
**<5 CTCs**	17/33 (51,5%)	12/33 (36,4%)	12/33 (36,4%)	13/37 (35,1%)	10/37 (27,0%)	7/37 (18,9%)	6/12 (50,0%)	7/12 (58,3%)	5/12 (41,7%)
**0 CTCs**	8/22 (36,4%)	6/22 (27,3%)	6/22 (27,3%)	8/30 (26,7%)	6/30 (20,0%)	3/30 (10,0%)	5/10 (50,0%)	6/10 (60,0%)	4/10 (40,0%)

*p* value: Baseline CellSearch vs IF: *0,03; **0,049.

**Table 4:** Detection of CTCs subpopulations with immunofluorescence in patients without detectable CTCs by CS.

Patient’s No	TTF^−^1^+^/CD45^−^	CD56^+^/CD45^−^	TTF^−^1^+^/CD56^+^	TTF^−^1^+^/EpCam^+^	CK^+^/EpCam^+^
1	56	44	44	0	0
2	13	15	13	0	0
3	3	1	1	0	0
4	38	12	10	0	0
5	19	3	2	0	0
6	169	69	53	0	0
7	6	0	0	0	0
8	2	0	0	0	0
