# Supplementary material for: TTF-1- and/or CD56-positive Circulating Tumor Cells in patients with small cell lung cancer (SCLC)
Source: Sci Rep. 2017 Mar 28;7:45351. doi: 10.1038/srep45351 (PMC5368597; doi:10.1038/srep45351)
Supplement: Supplementary Information [file srep45351-s1.pdf]

Supplementary Information

TTF-1- and CD56-positive circulating tumor cells in patients with small cell lung cancer (SCLC)

Ippokratis Messaritakis[1], Dimitris Stoltidis[1], Eleftheria- Kleio Dermitzaki[1], Fillipos Koinis[1], Athanasios Kotsakis[1,2], Eleni Lagoudaki[3], Eleni Politaki[1], Stella Apostolaki[1], John Souglakos[1,2], Vassilis Georgoulias[1]*

[1]Laboratory of Tumor Cell Biology, School of Medicine, University of Crete, [2]Department of Medical Oncology and [3]Pathology, University General Hospital of Heraklion, Crete, Greece

Supplementary Figure S1: Flow chart of the study.

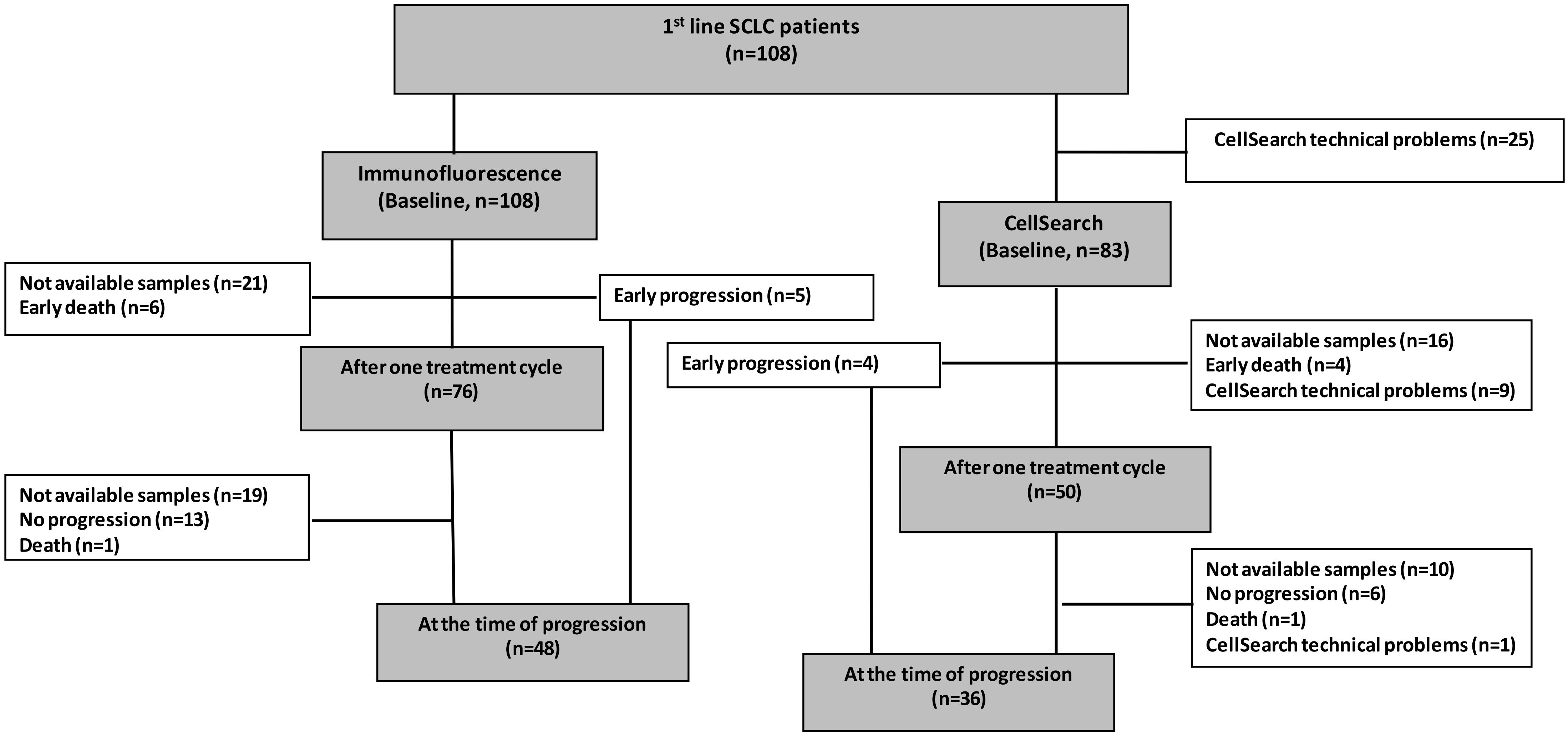

SCLC: Small cell lung cancer

Supplementary Table S1. Detection of TTF-1^{+}, CD56^{+} and TTF-1^{+}/CD56^{+} H209 cells

# Exp	TTF-1 (%)	CD56 (%)	TTF-1/CD56 (%)
**1**	80	70	73
**2**	90	100	63
**3**	83	96	66
**4**	84	78	64
**5**	89	82	72
**Mean (+/-)**	85±5	85±15	68±5

Supplementary Table S2. Detection of CTCs' subpopulations and clinical outcome

	Disease progression		
**CTCs subpopulation**	**Yes (N=89)**	**No (N=19)**	***p* value**
**CellSearch at baseline**	45 (54,2%)	5 (6,0%)	0,004
**TTF-1^{+}/CD45^{-} at baseline**	54 (50,0%)	12 (11,1%)	0,84
**CD56^{+}/CD45^{-} at baseline**	46 (42,6%)	9 (8,3%)	0,733
**TTF-1^{+}/CD56^{+} at baseline**	37 (34,3%)	9 (8,3%)	0,643
**CellSearch at baseline**	16 (29,0%)	0 (0,0%)	0,022
**TTF-1^{+}/CD45^{-} post 1st cycle**	26 (38,4%)	4 (5,9%)	0,223
**CD56^{+}/CD45^{-} post 1st cycle**	16 (23,5%)	6 (8,8%)	0,237
**TTF-1^{+}/CD56^{+} post 1st cycle**	16 (23,5%)	3 (4,4%)	0,477

Supplementary Table S3. Medians for survival time and overall comparisons

	PFS			OS		
	**Median months (95% CI)**		***p* value**	**Median months (95% CI)**		***p* value**
	**≥5 CTCs**	**<5 CTCs**		**≥5 CTCs**	**<5 CTCs**	
**CellSearch at baseline**	6,0 (5,4-6,7)	7,9 (5,7-10,1)	0,001	8,4 (7,0-9,8)	21,7 (15,6-27,7)	<0,001
**CellSearch post 1st cycle**	5,4 (3,2-7,5)	7,0 (6,8-7,2)	0,004	8,3 (6,2-10,3)	14,8 (10,3-19,3)	0,004
**CellSearch on PD**	-	-	-	9,1 (7,2-11,0)	19,3 (6,6-11,5)	0,021

Supplementary Table S4. Univariate and multivariate Cox Regression analysis

	Univariate Analysis				Multivariate Analysis			
	PFS		OS		PFS		OS	
	HR (95% CI)	Sig.	HR (95% CI)	Sig.	HR (95% CI)	Sig.	HR (95% CI)	Sig.
**PS (>2 vs 0-1)**	2,2 (1,5-3,4)	<0,001	2,7 (1,7-4,3)	<0,001	-	-	-	-
**ED vs LD**	3,2 (2,0-5,2)	<0,001	3,7 (2,1-6,5)	<0,001	-	-	-	-
**LDH (High vs Normal)**	2,1 (1,3-3,4)	,006	3,1 (1,7-5,9)	<0,001	-	-	-	-
**Liver Metastases (Yes vs No)**	2,2 (1,4-3,4)	<0,001	2,4 (1,5-3,8)	<0,001	-	-	-	-
**Bone Metastases (Yes vs No)**	2,4 (1,5-3,8)	<0,001	3,2 (1,9-5,3)	<0,001	-	-	-	-
**Response (PD vs SD vs CR/PR)**	1,2 (0,6-2,7)	<0,001	1,4 (0,6-3,3)	<0,001	-	-	-	-
**TTF-1 baseline (+ vs -)**	0,9 (0,6-1,4)	,780	1,3 (0,8-2,0)	,338	-	-	-	-
**CD56 baseline (+ vs -)**	1,1 (0,7-1,6)	,709	1,3 (0,8-2,0)	,339	-	-	-	-
**TTF-1/CD56 baseline (+ vs -)**	0,9 (0,6-1,3)	,554	0,9 (0,6-1,4)	,646	-	-	-	-
**TTF-1/CD56 on PD (+ vs -)**	-	-	1,9 (1,1-3.7)	,040	-	-	-	-
**CellSearch Baseline (≥5 vs <5 CTCs)**	2,4 (1,4-4,2)	,001	3,4 (1,8-6,3)	<0,001	1,9 (0,9-3,9)	0,048	-	-
**CellSearch Post 1st (≥5 vs <5 CTCs)**	1,9 (1,0-3,5)	,050	1,9 (1,0-3,6)	,058	-	-	-	-
**CellSearch on PD (≥5 vs <5 CTCs)**	-	-	2,1 (1,1-4,0)	,033	-	-	2,1 (0,9-5,3)	0,041
